# Um breve panorama sobre a Agenda 2030, as doenças crônicas não transmissíveis e os desafios de não deixar ninguém para trás

**DOI:** 10.1590/0102-311XPT139323

**Published:** 2024-08-23

**Authors:** Laurenice de Jesus Alves Pires, José Mendes Ribeiro, Marly Marques da Cruz

**Affiliations:** 1 Escola Nacional de Saúde Pública Sergio Arouca, Fundação Oswaldo Cruz, Rio de Janeiro, Brasil.

**Keywords:** Doenças Não Transmissíveis, Desenvolvimento Sustentável, Direitos Humanos, População Negra, População Indígena, Noncommunicable Diseases, Sustainable Development, Human Rights, Black People, Indigenous Peoples, Enfermedades no Transmisibles, Desarrollo Sostenible, Derechos Humanos, Población Negra, Pueblos Indígenas

## Abstract

Este ensaio traz uma reflexão teórica sobre os desafios para alcançar as metas dos Objetivos do Desenvolvimento Sustentável da Agenda 2030, considerando seu lema de “*não deixar ninguém para trás*”. Para exemplificar esses desafios, apresenta-se como pano de fundo as doenças crônicas não transmissíveis (DCNT), um dos principais temas da agenda da saúde global antes da pandemia de COVID-19, discutindo as dimensões políticas e econômicas que determinam sua presença e avanço global. Após um breve panorama sobre as DCNT, busca-se responder a três perguntas: em “Sem deixar ninguém para trás?”, elencamos alguns temas para refletir sobre como e quem tem ficado historicamente para trás, aprofundando um pouco mais os exemplos ao adentrar em “Quem tem ficado para trás no mundo?” e “Quem tem ficado para trás no Brasil?”. A partir de dados da literatura mais relevante e recente sobre o tema, apresentamos os desafios e alguns caminhos para não deixar ninguém para trás em um mundo em que o modo de produção tem historicamente vulnerabilizado alguns grupos sociais, com destaque para a população negra e a população indígena. Trazemos nas considerações finais a inspiração do ideograma Sankofa para lembrar que as respostas para o desenvolvimento sustentável que buscamos podem estar em algum lugar de nosso passado mais originário e tradicional, e que é preciso apostar na construção de novos caminhos a partir de outras epistemologias e cosmovisões presentes do outro lado da linha abissal.

## Abstract (en)

This essay provides a theoretical reflection on the challenges of meeting the Sustainable Development Goals of the 2030 Agenda, considering its motto of “*leave no one behind*”. To exemplify these challenges, we discuss noncommunicable diseases (NCDs), one of the main issues on the global health agenda before the COVID-19 pandemic, and the political and economic dimensions that determine their presence and global spread. After a brief overview of NCDs, the text seeks to answer three questions: In “Leaving no one behind?” we list some themes to reflect on how and who has historically been left behind, delving a little deeper into the examples in “Who has been left behind in the world?” and “Who has been left behind in Brazil?”. Using data from the most relevant and recent literature on the subject, we discuss the challenges and some ways to leave no one behind in a world where the mean of production has historically made some social groups vulnerable, especially black and Indigenous populations. In our final remarks, we draw inspiration from the Sankofa ideogram to remember that the answers to the sustainable development we seek may lie somewhere in our most primordial and traditional past. And that it is necessary to invest on building new paths from different worldviews and approaches to epistemology on the other side of the abyssal line.

## Resumen

Este ensayo aporta una reflexión teórica acerca de los desafíos para lograr las metas de los Objetivos de Desarrollo Sostenible de la Agenda 2030, teniendo en cuenta su lema “*no dejar a nadie atrás*”. Para ejemplificar estos desafíos, tomamos como telón de fondo las enfermedades crónicas no transmisibles (ECNT), uno de los principales temas de la agenda de salud global antes de la pandemia de COVID-19, discutiendo las dimensiones políticas y económicas que determinan su presencia y avance global. Después de un breve panorama de las ECNT, el texto busca responder tres preguntas: En “¿Sin dejar a nadie atrás?” enumeramos algunos temas para reflexionar sobre cómo y quién ha quedado históricamente atrás, profundizando un poco más los ejemplos al centrarse en “¿Quién ha quedado atrás en el mundo?” y “¿Quién ha quedado atrás en Brasil?”. Con base en datos de la literatura más relevante y reciente sobre el tema, presentamos los desafíos y algunas maneras de no dejar a nadie atrás en un mundo en el que el modo de producción ha vulnerabilizado históricamente a algunos grupos sociales, con énfasis en la población negra y en la población indígena. En nuestras consideraciones finales, nos inspiramos en el ideograma de Sankofa para recordar que las respuestas al desarrollo sostenible que buscamos pueden estar en algún lugar de nuestro pasado más original y tradicional. Y es necesario apostar por la construcción de nuevos caminos basados en otras epistemologías y cosmovisiones presentes al otro lado de la línea abisal.

## Um breve panorama sobre a Agenda 2030 e as doenças crônicas não transmissíveis

Estamos a seis anos de 2030, ano previsto para o alcance dos Objetivos do Desenvolvimento Sustentável (ODS), parte do mais atual e importante acordo global liderado pela Organização das Nações Unidas (ONU) em conjunto com todos seus Estados-membros. Para alcançar os 17 objetivos e 169 metas que integram a Agenda 2030, os Estados-membros se comprometeram a “*tomar medidas ousadas e transformadoras para promover o desenvolvimento sustentável nos próximos anos, sem deixar ninguém para trás*” ^1^.

Se, por um lado, os ODS dão continuidade aos esforços conjuntos iniciados com os objetivos de desenvolvimento do milênio para reduzir a pobreza mundial, por outro, marcam uma mudança fundamental ao ampliarem o foco de países pobres ou em desenvolvimento para todas as pessoas em todos os países ^2^. São orientados por uma agenda coletiva e ambiciosa ao tratar a economia, o social e o ambiente como dimensões estruturais para o desenvolvimento sustentável, de forma que o compromisso com o presente não comprometa as gerações futuras ^1^ ^,^ ^3^.

O alcance dos objetivos da Agenda 2030 e seu compromisso em não deixar ninguém para trás requer a revisão de um complexo emaranhado de relações sistêmicas no campo político-econômico, envolvendo países, governos, sociedade civil e grandes corporações internacionais e transnacionais. Essas relações orientam e são orientadas por macrodeterminantes sociais e econômicos que “*geram e reforçam hierarquias sociais que definem o poder, prestígio e acesso a recursos...*” ^4^ (p. 844), tanto de países quanto de indivíduos, impactando as formas de nascer, viver e morrer.

As doenças crônicas não transmissíveis (DCNT) - cânceres, doenças cardiovasculares, doenças respiratórias crônicas, diabetes e condições de saúde mental - são consideradas uma ameaça, sob amplos aspectos ^5^ ^,^ ^6^ ^,^ ^7^, para o desenvolvimento no século XXI, e, por isso, foram alçadas ao topo dos problemas da saúde global com sua entrada nos ODS. Elas compartilham do mesmo complexo emaranhado ao qual está submetida a Agenda 2030, uma vez que sua incidência e prevalência também são influenciadas por atores e poderes globais e locais, assim como pela globalização do modo de vida moderna.

As DCNT são responsáveis por 71% de todas as mortes no mundo, representando 41 milhões de mortes prematuras - entre 30-69 anos - em 2019 ^8^. As projeções sobre o custo global com as mortes prematuras até 2030 chegavam a US$ 47 trilhões, representando 75% do produto interno bruto (PIB) mundial em 2010. Prospecta-se que milhões de pessoas sejam empurradas para abaixo da linha da pobreza ^7^ ^,^ ^9^, uma vez que as condições de saúde e trabalho dos indivíduos, muitas vezes já vulnerabilizadas, são agravadas pelos custos do tratamento e dos cuidados necessários para o restabelecimento da saúde.

Reconhecendo o impacto social e econômico das DCNT, em 2011, a ONU e a Organização Mundial da Saúde (OMS) realizaram a primeira Reunião de Alto Nível para tratar sobre o tema.

No Brasil, o cenário não é diferente: 72% das causas de morte referem-se a esse conjunto de doenças ^10^. O país esteve na vanguarda das discussões, apresentando o Plano Nacional de DCNT para o período 2011-2022 ^11^. Na sequência, a OMS lançou o Plano de Ação Global para o Enfrentamento e Controle das DCNT, contemplando o período de 2013-2020 ^12^. Entre os objetivos centrais dos planos, constam como estratégias prioritárias a redução da carga de mortalidade prevenível e evitável por meio da colaboração multissetorial, a cooperação em nível nacional, regional e global, e o enfrentamento do consumo de tabaco, da alimentação não saudável, do consumo nocivo de álcool, da inatividade física e da poluição do ar, por serem considerados os fatores de risco comuns a essas doenças.

Os esforços para o enfrentamento das DCNT ganharam força com sua entrada nos ODS da Agenda 2030, integrando a meta 3, de saúde e bem-estar, mais especificamente a meta 3.4: reduzir em um terço a mortalidade prematura via prevenção e tratamento, e promoção da saúde mental e do bem-estar ^13^. Em nível regional, com a Agenda 2063 do continente africano, integram a meta 3: cidadãos saudáveis e bem nutridos ^14^.

O enfrentamento das DCNT não se limita aos fatores de risco - assim como as estratégias para a efetivação do compromisso de não deixar ninguém para trás, é necessário superar os determinantes estruturais comuns. Considerando esse cenário, as DCNT serão discutidas neste ensaio menos por seu aspecto epidemiológico e mais pelas implicações político-econômicas que as promovem a uma realidade global. Buscaremos exemplificar como alguns grupos sociais têm sido impactados por determinantes sociais e econômicos como a globalização, raça/etnia, gênero, trabalho, renda e sistema de saúde, sendo alijados dos benefícios sociais, políticos, econômicos, culturais e ambientais resultantes do projeto de desenvolvimento ao qual todos os países têm sido submetidos, embora com diferentes níveis de acesso aos bens produzidos.

Diante deste cenário, apresentam-se as reflexões: como será possível não deixar ninguém para trás? Quem tem ficado para trás? Como é possível, a partir do aprendizado do caminho trilhado, construir alternativas eficazes que não deixem ninguém para trás no Brasil e no mundo?

## Sem deixar ninguém para trás?

Assumir o compromisso global de “*não deixar ninguém para trás*” requer coragem! Coragem para admitir que o caminho trilhado até o momento deixou muitos para trás, produzindo mortes evitáveis, exclusões e iniquidades em vários campos da vida econômica, social e ambiental. Coragem em relação ao futuro, para apostar na construção de modelos sociais que tenham como princípio não deixar ninguém para trás, ou seja, que sejam capazes de reduzir as iniquidades que historicamente têm produzido mortes e sofrimentos evitáveis para uma grande parcela da população mundial, distribuindo entre eles os benefícios alcançados com o desenvolvimento e produzindo saúde mental e bem-estar. Para tanto, é preciso fazer valer os princípios da universalidade e da equidade e cumprir antigos e novos acordos estabelecidos local e globalmente.

O compromisso assumido com a Agenda 2030 pela ONU e suas agências, os 193 Estados-membros e empresas, organizações da sociedade civil e outros atores sociais nacionais e internacionais, com ou sem fins-lucrativos, aponta para uma oportunidade promissora de não deixar ninguém para trás. Está alinhado com importantes compromissos globais, como a *Declaração Universal dos Direitos Humanos* ^15^, a Conferência de Alma-Ata ^16^, a *Carta de Ottawa* ^17^, a Convenção Internacional sobre a Eliminação de todas as Formas de Discriminação Racial ^18^ e a *Declaração sobre os Povos Indígenas* ^19^, que também dialogam com a Constituição da OMS ^20^ e a *Carta das Nações Unidas* ^21^.

Mas como avançar do compromisso teórico ao compromisso real, de modo a não deixar ninguém para trás? De qual realidade partimos para pensar em estratégias para atingir esse objetivo?

Embora sejam inegáveis os avanços tecnológicos, econômicos e sociais trazidos pela globalização, seus resultados não têm alcançado a todos. Ao contrário, muitos têm ficado para trás no acesso a seus benefícios. O anúncio de que todos viveriam integrados, em uma aldeia global, tentou esconder o que Santos ^22^ chamou de globalização como perversidade: a perversidade do acesso desigual aos benefícios da globalização, da priorização dos interesses econômicos sobre os sociais, responsáveis por profundas iniquidades, entre e dentro dos países. Esse é um cenário anteriormente analisado por Castro ^23^, definido como progresso de fachada, por ser resultante de políticas orientadas por interesses privados, limitadas aos setores mais rendosos das sociedades e voltadas para a manutenção da riqueza de países ricos a partir da exploração da riqueza dos países pobres. Um progresso para poucos, nas palavras do autor.

Essa perversidade se concretiza no fato de que 1% das pessoas mais ricas globalmente concentram a mesma riqueza que 60% de toda a população mundial ^24^, ou que 828 milhões de pessoas no mundo (8% da população) não tinham nada para comer ou alimentar os seus no ano de 2021 ^25^.

Essas e outras iniquidades foram intensificadas pela emergência da pandemia de COVID-19 - que teve início declarado em março de 2020 e fim em maio de 2023 -, contexto no qual se pôde observar o aumento do consumo de alimentos ultraprocessados, do sedentarismo e a redução de atividade física ^26^, com destaque entre pessoas com DCNT, com mais idade e menor escolaridade, assim como maior adesão ao distanciamento social por pessoas com uma ou mais DCNT ^27^. Ademais, ressaltam-se as insuficientes respostas políticas e econômicas dadas no âmbito da governança local e global. Algumas dessas questões, porém, precedem esse momento pandêmico, pois conformam o modelo de desenvolvimento hegemonicamente excludente, sustentado pelo colonialismo e pela colonialidade, tendo a exploração do trabalho e das riquezas, a naturalização das hierarquias territoriais, étnico-raciais, de gênero, culturais e epistêmicas e a (re)produção das relações de poder como centrais para sua manutenção ^28^. Crianças, mulheres e populações e países mais pobres sofrem mais intensamente o impacto negativo dessa forma de globalização e desenvolvimento ^29^ ^,^ ^30^ ^,^ ^31^ ^,^ ^32^.

Nos países pobres, somam-se aos desafios sanitários os desafios políticos, econômicos, sociais e ambientais dos quais eles historicamente não têm conseguido se desvencilhar ^33^ ^,^ ^34^, visto que suas políticas sociais e econômicas são controladas por países ricos e poderosos, dos quais os países pobres se encontram dependentes e *ad aeternum* ^35^ devedores.

Nesse contexto, os países pobres precisam lidar com uma tripla carga de doenças: as infecciosas e parasitárias, as crônicas e as causas externas, além da tríade - obesidade, subnutrição e mudança climática - batizada de sindemia global ^36^ ^,^ ^37^. A situação é agravada pelo avanço do envelhecimento da população mundial, que tem projeção de dobrar até 2050 e triplicar até 2100, chegando a 3,1 milhões de pessoas, sendo os países de baixa e média renda os locais nos quais viverá 80% dessa população ^38^ ^,^ ^39^. Não obstante, é nesses países que se encontram grande parte dos sistemas de saúde fragilizados e distantes dos princípios de universalidade e equidade necessários para assegurar a saúde como um direito humano fundamental.

A cobertura universal de saúde é a grande aposta global para garantir o cuidado integral à saúde sem riscos financeiros. A meta 3.8 consiste em “*atingir a cobertura universal de saúde, incluindo a proteção do risco financeiro, o acesso a serviços de saúde essenciais de qualidade e o acesso a medicamentos e vacinas essenciais seguros, eficazes, de qualidade e a preços acessíveis para todos*”. Um de seus indicadores é o índice de cobertura universal, que vai de 0 a 100. O monitoramento global dos ODS ^40^ mostra que, em 2019, o grupo de países de baixa renda alcançou 42 pontos do índice, sendo relatados “desafios importantes persistentes” como justificativa para o resultado. Por sua vez, os países de alta renda alcançaram 83 pontos, sendo relatados como países que estavam “no caminho certo”, sem considerar as relações histórico-políticas de dependência e a assimetria de poderes que explicam esses resultados ^41^.

O relatório do Grupo de Trabalho (GT) de Especialistas para Inclusão de Pessoas de Descendência Africana, ao analisar a meta 3.8, relatou que “*mesmo em países muito desenvolvidos, as pessoas de ascendência africana enfrentam barreiras para acesso aos cuidados de saúde*” ^42^. Barreto ^43^ (p. 203), ao analisar os desafios globais contemporâneos na saúde global, lembra que pesquisas da saúde pública e da epidemiologia acumulam evidências de que “*...a ocorrência das mais diversas doenças e problemas de saúde se agrava* (...)*, entre os mais pobres, entre grupos étnicos minoritários ou grupos que sofrem qualquer tipo de discriminação*”.

Giovanella ^44^, ao analisar os desafios dos sistemas de saúde na América Latina e no Caribe, observa como características a segmentação da cobertura e a privatização do financiamento e da prestação de serviços. Os prestadores privados, embora atendam mais prontamente e ofereçam cuidados mais personalizados - por atenderem uma parcela menor da população -, “*...violam mais frequentemente os padrões de boa prática médica e têm resultados inferiores em saúde dos pacientes*” ^44^ (p. 236). A fragilidade dos sistemas públicos de saúde e o lento avanço da cobertura universal de saúde representam um agravo central para a oferta de cuidados em saúde em muitos países, uma vez que quanto mais pobre é um país, mais desafios são enfrentados para lidar com a transição epidemiológica ainda em curso. Embora as doenças transmissíveis ainda sejam muito incidentes e prevalentes, as doenças crônicas já são uma realidade que muitas vezes acometem os mesmos indivíduos, implicando em maior agilidade e efetividade em sistemas de saúde pouco estruturados ou já fragilizados ^33^ ^,^ ^43^.

Embora a alimentação seja essencial no processo de saúde, a produção alimentícia definitivamente se afastou da concepção de alimento como direito humano, afirmando-se como alimento-mercadoria ^45^. Ela é orientada por uma lógica imperialista, centrada na monocultura, na transgenia, no uso de adubos químicos e agrotóxicos, na monopolização das patentes e na apropriação privada da terra, viabilizada pelo cerceamento de países pobres a meros exportadores de produtos primários ^45^ ^,^ ^46^.

Esse cenário é agravado pelas condições do trabalho assalariado, fetichizado, alienado e precarizado ^47^ ^,^ ^48^, frequentemente mal ou não remunerado, em especial no caso das mulheres ^49^, assim como pelo subemprego e desemprego sistêmicos aos quais está submetida grande parcela da população mundial. Esse panorama configura solo fértil para a produção e reprodução das condições que geram as DCNT, reafirmando a necessidade de entendimento do processo saúde-doença a partir do conceito de determinação da saúde ^4^. Nesse contexto, as estratégias voltadas para o enfrentamento dos fatores de risco, com políticas pontuais e práticas individuais, biológicas e biomédicas, ligadas ao lado perverso da globalização - que padroniza o modo de nascer, viver, produzir, adoecer e morrer ^50^ e produz doenças e mal-estar -, oferecem respostas circunscritas, que tendem a deixar muitos para trás.

Embora o Plano Global de Enfrentamento das DCNT seja ousado ao tornar público o comprometimento em não deixar ninguém para trás, e apesar de importantes avanços na condição de saúde e acesso das populações no cenário internacional e nacional, os desafios para tornar esse compromisso real exigem mudanças estruturais que enfrentem os macrodeterminantes que estão na raiz do avanço das DCNT ^43^, muitos deles fora do controle direto da saúde ^51^. São requeridas mudanças que problematizem a desigual correlação de forças que mantém a relação de dominação histórico-estrutural de países do Norte Global sobre países do Sul Global ^28^. Adicionalmente, são necessárias transições que centralizem o capitalismo global como a principal determinação social e econômica a ser revista ^51^.

Respostas efetivas que tenham como princípio não deixar ninguém para trás no campo da saúde e do bem-estar não deveriam passar ao largo: (i) do fortalecimento dos sistemas de proteção social universais e gratuitos; (ii) do combate ao pensamento abissal ^52^; (iii) do equilíbrio da correlação de forças entre os países, de forma a garantir cooperações multilaterais; (iv) da discussão sobre a legalidade, legitimidade e eticidade da dívida externa dos países ^35^; e (v) do respeito a outros saberes, práticas e modos de viver e suas cosmovisões ^35^ ^,^ ^53^.

## Quem tem ficado para trás no mundo?

Na Declaração Ministerial de Alto Nível produzida em função da Reunião Política de Alto Nível sobre o Desenvolvimento Sustentável realizada em julho de 2022 ^32^, ministros reafirmaram que “*os países menos desenvolvidos, como o grupo mais vulnerável de países, precisam de apoio global reforçado para superar os desafios estruturais, o impacto devastador recente da pandemia de COVID-19 e outros obstáculos que esses países enfrentam na implementação da Agenda 2030*”.

No decorrer da pandemia de COVID-19, o atendimento às pessoas com DCNT na região das Américas foi reduzido ou interrompido em muitos países, em função do fechamento dos serviços para o redirecionamento da força de trabalho para atendimento aos pacientes acometidos pelo novo vírus ^54^, assim como pelo afastamento dos pacientes, em especial os idosos, para adesão ao distanciamento por medo de infecção ^10^ ^,^ ^55^. Esse contexto ficou ainda mais tenso com a escassez do acesso a vacinas e equipamentos de proteção individual, motivada pela ganância de países ricos ^56^.

Durante o Fórum Político de Alto Nível sobre Desenvolvimento Sustentável, a organização não governamental (ONG) Major Group, que representa globalmente algumas organizações que trabalham em prol do alcance dos ODS, apresentou seu Documento de Posição de 2022 ^32^, informando que mulheres, jovens, indígenas e pessoas com deficiência são os grupos identificados como mais propensos a serem deixados para trás. A ONG destacou como preocupantes as situações de exploração de mulheres e crianças, o retrocesso das ações em direitos humanos, os conflitos regionais e nacionais, as ameaças à biodiversidade e a divisão digital entre países ricos e pobres, rurais e comunidades remotas.

Outros estudos internacionais evidenciam mais detalhadamente quem tem ficado para trás e como: (i) o acesso à água potável é uma realidade para 74% da população mundial, sendo 96% na Europa e na América do Norte e 30% na África Subsaariana ^57^; (ii) 0,4% das pessoas que vivem com US$ 1,90 por dia estão em países de alta renda e 41,7% em países de baixa renda; e (iii) 89,8% da população que usa a internet está em países de alta renda, na África Subsaariana são somente 28,4% ^58^.

O GT de Especialistas para Inclusão de Pessoas de Descendência Africana analisou cada meta dos ODS, indicando desafios enfrentados e recomendações para inclusão da população negra. Em relação à meta 3.4, o GT “*encoraja os Estados a introduzirem programas especificamente designados para pessoas de descendência africana visando reduzir a mortalidade prematura por DCNT*” ^42^. Segundo o documento, o GT apresenta argumentos convincentes em matéria de direitos humanos que justificam a necessidade de direcionar especial atenção às pessoas de ascendência africana como um dos grupos populacionais que enfrentam formas múltiplas e combinadas de discriminação e que devem ser consideradas prioritárias para acabar com as desigualdades e a discriminação, para “*não deixar ninguém para trás*” e para “*chegar primeiro aos mais desfavorecidos*” ^42^.

A população indígena mundial é composta por aproximadamente 476 milhões de pessoas (6% da população mundial). No entanto, aproximadamente 19% desse grupo está em situação de extrema pobreza e tem a expectativa de vida 20 anos menor que pessoas não-indígenas. Em muitos países, a população indígena não integra as estatísticas nacionais, uma vez que não são produzidos dados desagregados para os monitoramentos nacionais, inclusive dos ODS. Somente 10% dos territórios indígenas são legalmente reconhecidos, embora protejam 80% da biodiversidade mundial ^59^ ^,^ ^60^. Dada a importância desse grupo para o desenvolvimento sustentável, Yap & Watene ^61^ (p. 6) reclamam a presença da cultura entre os objetivos dos ODS, lançando a pergunta: “*se a cultura não aparece como uma dimensão e, portanto, um objetivo do desenvolvimento sustentável, como é possível que as aspirações indígenas tenham sido incluídas?*”.

A ausência dos grupos em situação de vulnerabilidade nos espaços de construção e discussão dos acordos, metas e indicadores globais ou locais também é uma forma de deixá-los para trás.

“*...as diferentes concepções destas dimensões* [dos ODS] *nas diferentes culturas significam que, no processo de obtenção de um consenso universal, as prioridades e os pontos de vista de alguns grupos não serão levados em conta, especialmente os que se encontram à margem. Se esses pontos de vista não fizerem parte da estrutura de prestação de contas e monitoramento, ou seja, se forem invisíveis, como poderemos saber se alcançamos um futuro em que ninguém é deixado para trás? Mais especificamente, quais serão as prioridades que se tornarão visíveis? As dimensões complexas e ‘demasiado difíceis de medir’ serão deixadas de lado?*” ^61^ (p. 5).

A população e os países mais pobres, grupos sociais em maior condição de vulnerabilidade como mulheres, jovens e pessoas com deficiência e os grupos racializados, com destaque para a população afrodescendente e a população indígena, são aqueles que têm enfrentado maiores desafios para acessar os bens socialmente produzidos com o desenvolvimento global, tendo assim maior risco de (continuar a) ficar para trás. Por isso, o enfrentamento das DCNT requer ações que extrapolem o campo *stricto sensu* da saúde, assim como o foco na meta 3. Todas as metas da Agenda 2030 são importantes para o pleno alcance da meta 3 e vice-versa. É nessa relação intrínseca entre as metas dos ODS que se apresenta seu potencial ousado de transformação, além de seu risco: para não deixar ninguém para trás, é necessário priorizar os grupos marginalizados nas estratégias políticas, assim como incluí-los em todas as etapas, da construção à implementação das ações.

## Quem tem ficado para trás no Brasil?

Similarmente ao que tem acontecido no mundo, no Brasil, a população negra, jovem e indígena tem ficado para trás. Embora 56,1% da população brasileira tenha se autodeclarado como preta ou parda, esse grupo está sub-representado nos cargos de poder (29,9%) e na representação política (24,4%), e sobre-representado na força de trabalho desocupada (64,2%), subutilizada (66,1%) e no grupo de pessoas que vive abaixo da linha de pobreza (32,9%) ^62^.

Em 2019, a taxa de violência letal contra pessoas negras foi 162% maior do que entre pessoas não negras, sendo que muitas dessas mortes ocorreram em ações policiais ^63^. Nessa década, foi observado o aumento de 1,6% dos homicídios entre pessoas negras e a redução de 33% entre pessoas não negras ^64^. Jovens negros representam 61% das pessoas entre 15 e 29 anos no Brasil. Eles estão sub-representados nas classes A e B, encontrando-se em situação de maior vulnerabilidade e sendo o principal alvo da violência. No biênio 2017-2018, jovens de 15 a 19 anos na área rural tinham 14,6% mais probabilidade de entrar na extrema pobreza ^65^. O Governo Federal instituiu o GT interministerial para elaboração da proposta do Plano Juventude Negra Viva ^66^, envolvendo 16 ministérios, dada a complexidade, gravidade e urgência da situação.

Os povos indígenas também têm ficado para trás. Mais de 800 indígenas da etnia Yanomami foram internados em situação de desnutrição grave em janeiro de 2023, após vários pedidos de socorro terem sido feitos durante a gestão do Presidente Jair Bolsonaro, sem sucesso, incentivando um cenário em que a população de garimpeiros está quase igual à população nativa, com 30 mil e 20 mil habitantes, respectivamente ^67^.

Os ODS são a aposta global e, em muitos casos, local, para amenizar a situação de pobreza à qual alguns grupos estão submetidos. No entanto, o VI Relatório Luz, publicado por um GT formado por 48 organizações e 101 especialistas que monitoram as 169 metas do Painel ODS Brasil ^68^, registrou que, em 2022: 65,5% das metas estão em retrocesso; 6,5% permanecem ou entraram em estagnação, 8,3% ameaçadas, 14,3% em progresso insuficiente, e 84,8% não dispõem de informação. Somente para a meta 15.8 foi registrado progresso satisfatório: “*até 2020, implementar medidas para evitar a introdução e reduzir significativamente o impacto de espécies exóticas invasoras em ecossistemas terrestres e aquáticos, e controlar ou erradicar as espécies prioritárias*” ^69^. A meta 3.4 - reduzir em um terço a mortalidade prematura via prevenção e tratamento, e promoção da saúde mental e do bem-estar - passou de estagnada (2021) para ameaçada (2022) ^69^. Vale destacar que, no cenário brasileiro, a situação foi agravada por uma gestão presidencial que interrompeu o monitoramento dos ODS, reduziu o orçamento para políticas públicas sociais, eliminou espaços de participação popular e apagou dados oficiais, como apresentado no Relatório Luz de 2022.

Essa gestão produziu, entre 2021 e 2022, mais de 14 milhões de brasileiros famintos! Totalizaram-se 33 milhões de brasileiros (15,5% da população) que não tinham o que comer. Essa realidade é ainda mais intensa para quem vive no Norte (26%) e Nordeste (21%), quem solicitou e não recebeu o auxílio emergencial (63%), quem não tinha água em casa (42%), quem é preto e pardo (18,1%) e quem é mulher (19,3%) ^25^. As principais ações políticas econômicas adotadas durante essa gestão foram deliberadamente de encontro com a promessa de não deixar ninguém para trás, comprometendo as estratégias de enfrentamento das DCNT e o compromisso da Agenda 2030 de não deixar ninguém para trás. Para o grupo populacional de pretos e pardos, observou-se um aumento de 60% das pessoas que convivem com a fome, enquanto entre brancos esse aumento foi de 34,6%, de acordo com o 2º Inquérito de Insegurança Alimentar ^70^.

O estudo de Malta ^10^, com base no *Sistema de Vigilância de Fatores de Risco e Proteção para Doenças Crônicas por Inquérito Telefônico* (Vigitel), realizado com 45.448 adultos, nas 27 capitais do Brasil em 2012, mostra as diferenças dos fatores de risco em razão da raça/cor (Quadro 1).

**Quadro 1:** Fatores de risco das doenças crônicas não transmissíveis, de acordo com o *Sistema de Vigilância de Fatores de Risco e Proteção para Doenças Crônicas por Inquérito Telefônico* (Vigitel), em todas as capitais brasileiras, por raça/cor, 2012.

	MULHERES PRETAS	HOMENS PRETOS	MULHERES PARDAS	HOMENS PARDOS
Atividade física	Maior prevalência em atividades físicas no trabalho e na atividade doméstica, resultantes de trabalhos menos qualificados			
Alimentação	Maior consumo de carnes com gorduras		Menor consumo de frutas e hortaliças	
Hipertensão arterial		Maior taxa de hipertensão arterial		
Consumo de tabaco			Menor razão de prevalência de fumo e consumo de 20 cigarros diários	
Consumo de álcool	Menor prevalência de consumo de álcool e direção			

Fonte: elaboração própria a partir de Malta et al. ^74^.

Embora a população afrodescendente e indígena esteja sendo deixada para trás, no mundo e no Brasil, vale destacar o trabalho de organizações da sociedade civil (OSC) na defesa da garantia de direitos fundamentais de populações ou grupos expostos à situação de maior vulnerabilidade, como as nacionais Coalizão Negra por Direitos (https://coalizaonegrapordireitos.org.br/), que defende os direitos da população negra, a Articulação dos Povos Indígenas do Brasil (https://apiboficial.org/), que defende os direitos da população indígena, e o Grupo de Trabalho da Sociedade Civil para a Agenda 2030 (https://gtagenda2030.org.br/), que monitora os ODS no Brasil. Em relação às organizações internacionais, destacam-se as organizações NGO Major Group (https://www.ngomg.org/), Women in Global Heath (https://womeningh.org/about/), que defende a igualdade de gênero na saúde global, e a Global Health Watch (https://phmovement.org/global-health-watch), uma plataforma de resistência para o domínio neoliberal na saúde, entre tantas outras organizações e temas.

No México, um grupo de trabalho composto por OSC produziu o Informe Luz 2021 ^2^, que aponta para desafios nacionais de inserção dos ODS na estrutura executiva e legislativa do país, nos níveis nacional e local, com a criação de um Conselho Nacional para a Agenda 2030 e Comitês de Trabalho, mecanismos de participação e incidências das OSC e Cooperação Internacional, com ampla difusão dos compromissos assumidos para a população.

No Brasil, o V Relatório Luz da Sociedade Civil, de 2021 ^71^, aponta que: “*...há maior adesão à Agenda 2030 pelos espaços subnacionais, onde comissões municipais e estaduais ODS avançam na localização da Agenda 2030; há também a iniciativa do Poder Judiciário de indexar sua base de dados (de 80 milhões de processos) aos ODS e um projeto de lei que nacionaliza a Agenda 2030, o PL 1308/2021, foi pautado no Congresso Nacional pela Frente Parlamentar Mista de Apoio aos ODS*”.

O relatório da pesquisa global sobre DCNT em 2019, feito pela OMS ^72^, informa que 95% dos países tinham uma unidade ou departamento de DCNT, 74% incluíram as DCNT em seus planos nacionais e um terço havia implementado políticas para reduzir o impacto do marketing de comidas não saudáveis para crianças ou para reduzir o consumo de açúcar e sal. A edição 2022 do Monitor de Progresso das DCNT ^73^ indica que 126 dos 193 países do mundo estabeleceram metas nacionais de DCNT seguindo as orientações da OMS e, mesmo com a pandemia de COVID-19, 77 países alcançaram plenamente mais indicadores do que foi observado no relatório anterior.

Esses exemplos evidenciam um esforço para avançar no sentido do compromisso assumido pelos países, representando algum nível de envolvimento com o compromisso de não deixar ninguém para trás. No entanto, o alcance das metas dos ODS, no âmbito nacional ou global, requer transformações que ultrapassem o campo retórico e se efetivem no campo prático, político e econômico. São necessários acordos baseados em uma outra ética política, econômica e cultural, construída sob a égide da equidade e do respeito aos direitos humanos fundamentais.

## Considerações finais para não deixar ninguém para trás!

O alcance dos objetivos da Agenda 2030 requer o enfrentamento de iniquidades que historicamente têm excluído uma grande população mundial. Em um cenário que cotidianamente deixa pessoas para trás, é preciso criar, revisitar e/ou personalizar estratégias políticas capazes de produzir e estimular novas realidades. Logo, a reflexão sobre os desafios para alcançar as metas dos ODS faz-se urgente e atual, pois as DCNT continuam a ser um importante problema de saúde pública no Brasil e no mundo. A pandemia de COVID-19 complexificou o alcance das metas dos ODS como um todo, das metas relacionadas às DCNT e, mais especificamente, dos cuidados às DCNT.

Embora os macrodeterminantes sociais da saúde coloquem em xeque o alcance das metas dos ODS, eles representam o grande acordo global na área da saúde que faz com que os governos tenham uma agenda de trabalho orientada por um mesmo tema, que só poderá ser efetivada com a participação de todos em todos os níveis. O alcance dessas metas aponta, de fato, para um mundo melhor.

No entanto, a não integração dos conhecimentos seculares sobre o modo de produção e reprodução sustentável dos povos originários tradicionais e do Sul Global e a ausência de ruptura com esse modelo de desenvolvimento baseado na exploração e em hierarquias raciais, de gênero e territoriais, são empecilhos estruturais para que se alcance um mundo sustentável e equitativo. O modelo de desenvolvimento atual já deu provas de que não é capaz de produzir sustentabilidade para todos, assim como o planeta vem demonstrando que não é possível ser sustentável somente para alguns.

Talvez por viver longamente em cenários de intensas iniquidades, já produzimos declarações, cartas, acordos e evidências demasiadamente potentes para apontar caminhos que pretendem não deixar ninguém para trás. No entanto, é preciso sair do campo da retórica e aplicá-los no campo prático. É preciso não deixar ninguém para trás como um princípio ético-epistemológico-científico-social-político-relacional, e apostar na construção de novos caminhos a partir de outras epistemologias e cosmovisões presentes do outro lado da linha abissal. A globalização é uma potencial importante aliada nesse processo, se, como dito por Santos ^22^ (p. 20), for orientada por bases técnicas e “*postas a serviço de outros fundamentos sociais e políticos*”.

O ideograma Sankofa é representado por uma ave que tem os pés voltados para frente, a cabeça para trás e segura um ovo na boca. Ele representa em imagem um provérbio tradicional dos povos de língua Akan da África Ocidental, Gana, Togo e Costa do Marfim, traduzido como aprender com o passado para construir o futuro ou voltar para buscar o que esqueceu.

Pensar em desenvolvimento sempre remete ao futuro, ao progresso. Contudo, talvez as respostas para o desenvolvimento sustentável e capaz de não deixar ninguém para trás estejam em algum lugar de nosso passado mais originário e tradicional. A boa notícia é que temos todas as ferramentas para (re)encontrá-las.
